# Relationship between growth mindset and self-rated health in Chinese college teachers: sequential mediating effect of health self-efficacy and physical exercise

**DOI:** 10.3389/fpubh.2025.1421319

**Published:** 2025-07-22

**Authors:** Tao Yu, Change Zeng, Lidong He, Hu Ying, Jie Liu, Yuzhen Wu, Jiayuan Li, Yaxing Guo, Yanjun Chen, Xiaofu Pan

**Affiliations:** ^1^College of State Governance, Southwest University, Chongqing, China; ^2^College of International Education, Sichuan International Studies University, Chongqing, China; ^3^School of Ethnology and Sociology, Minzu University of China, Beijing, China; ^4^Chongqing Vocational Institute of Engineering, Chongqing, China

**Keywords:** growth mindset, health self-efficacy, physical exercise, self-rated health, self-regulation theory

## Abstract

**Background:**

Chinese college teachers’ health issues have become increasingly significant, making it crucial to promote their physical health. Based on self-regulation theory, this study aims to explore the relationship and underlying mechanisms between Chinese college teachers’ growth mindset of health and their self-rated health.

**Methods:**

The study involved 456 Chinese college teachers (213 males, 243 females) who filled out a questionnaire measuring their growth mindset of health, health self-efficacy, physical exercise, and self-rated health.

**Results:**

The correlation analysis revealed positive links among Chinese college teachers’ growth mindset of health, health self-efficacy, physical exercise, and self-rated health. Further regression analysis indicated that their growth mindset of health positively influenced self-rated health, mediated by health self-efficacy and physical exercise.

**Conclusion:**

This study discovered a positive link between Chinese college teachers’ growth mindset of health and their self-rated health. It also emphasized the sequential mediating effect of health self-efficacy and physical exercise in this correlation, offering theoretical grounding and valuable insights for promoting college teachers’ physical health.

## 1 Introduction

The health issues of Chinese people have escalated in recent decades, with health risks steadily rising. Chinese college teachers, facing significant work stress, are particularly vulnerable to health problems and represent a high-risk group (1). Studies showed that their health indicators were often worse than their peers and worsening yearly (2). For instance, hypertension, hyperglycemia, thyroid nodule rates, and breast nodule rates had all increased (2). Another investigation also found high rates of hyperlipidemia, overweight/obesity, hypertension, and hyperglycemia among them (3). These alarming trends underscore the urgent need to address the health concerns of Chinese college teachers.

College teachers’ health status may be influenced by their health-related beliefs, specifically their health mindset. This mindset reflects whether they believe their health is unchangeable (fixed mindset) or can be improved through effort (growth mindset) (4). Such beliefs shape individuals’ motivations, behaviors, and performance (5, 6). Therefore, growth mindset of health may enhance health outcomes by fostering positive health motivations and behaviors. Our study, guided by self-regulation theory (7), aims to explore the link between growth mindset of health and self-rated health among Chinese college teachers, while also exploring the mediating roles of health self-efficacy and physical exercise.

### 1.1 Growth mindset and physical health

Mindset, refers to individuals’ implicit beliefs about whether their intelligence can change (5). This implicit theory was later renamed mindset theory by Dweck to promote its widespread understanding and intervention programs (8). This framework expanded the concept of plasticity beyond intelligence to include attributes like ability, morality, personality (6). Intervention promoting a growth mindset, which taught that people could change through effort and strategies, resulted in adolescents experiencing fewer physical illnesses compared to their peers over an eight-month period (9). This provided evidence for the positive association between growth mindset of personality and physical health.

In recent years, growth mindset has increasingly been applied to health research (10). Individuals with a growth mindset of health believe health can be improved through effort, whereas those with a fixed mindset of health see health as unchangeable (4). Evidence suggested a positive link between a growth mindset of health and improved health outcomes. For instance, college students with a growth mindset of health had a lower body mass index, indicating better health (4). In summary, we propose Hypothesis 1: growth mindset of health is positively correlated with the self-rated health in Chinese college teachers.

### 1.2 Mediating effect of physical exercise

The growth mindset of health may foster healthier behaviors. For instance, during the COVID-19 pandemic, those with a growth mindset were more likely to engage in protective behaviors like wearing masks, maintaining social distance, frequent handwashing, and sanitizing their phones and surroundings (11, 12). Conversely, individuals with a fixed mindset reported less adherence to these practices. Consequently, we suggest that college teachers with growth mindset of health are more likely to embrace health-promoting behaviors.

Specifically, we focus on physical exercise as a key health-promoting behavior, as numerous studies support its benefits for physical health. Growth mindset, according to self-regulation theory, could enhance self-regulation processes (7), including goal setting, monitoring, and achievement. Within this framework, physical exercise represents a crucial aspect of health goal achievement. Preliminary evidence suggested a positive link between a growth mindset of health and increased physical exercise (10). For instance, college students with growth mindset engaged more in both light and intense exercise (4), and exercised more frequently and regularly than those with fixed mindset (13). Furthermore, individuals with growth mindset persisted in exercise despite challenges (14), promoting long-term health goals. Therefore, we propose hypothesis 2: the positive correlation between growth mindset of health and self-rated health in Chinese college teachers is mediated by physical exercise.

### 1.3 Mediating effect of health self-efficacy

We aim to determine if the positive association between growth mindset of health and both physical health and physical exercise is mediated by health self-efficacy. In other words, we inquire whether growth mindset indirectly affects college teachers’ physical health through health self-efficacy and physical exercise. Self-efficacy represented individuals’ confidence in executing a given task or behavior (15). In the health context, this specifically pertained to health self-efficacy, reflecting individuals’ belief in their ability to manage their health (16). Health self-efficacy, shaped by various individual factors (17), could be positively influenced by growth mindset of health, which fostered positive health beliefs and enhances self-efficacy (10). Those who believed health was changeable tended to have higher nutritional and exercise self-efficacy, leading to healthier eating and overcoming exercise obstacles (14). Hence, we suggest that college teachers with growth mindset of health possess greater health self-efficacy.

Health self-efficacy, crucial for college teachers’ physical health, reflected their perceived ability for self-regulation (18). This perception is likely to foster positive health-related outcomes. Researches have confirmed a positive correlation between health self-efficacy and physical health. Adults with higher health self-efficacy enjoyed better health-related quality of life (19) and maintained lower body mass index (20), indicators of superior physical health. Consequently, we suggest that college teachers with stronger health self-efficacy enjoy a higher level of physical health. Accordingly, we propose hypothesis 3: the positive correlation between growth mindset of health and self-rated health in Chinese college teachers is mediated by health self-efficacy.

The positive relationship between health self-efficacy and physical health may be attributed to physical exercise. Prior researches indicated that individuals with higher health self-efficacy tended to exhibit more health-promoting behaviors. For instance, they demonstrated increased self-precautionary practices during the COVID-19 pandemic (21) and reduced suicidal behavior among cancer patients (22). In daily life, these individuals also adopted healthier lifestyles (23) and engaged in more health-promoting behaviors, including physical exercise (24). Based on these findings, we suggest that college teachers with stronger health self-efficacy spend more time exercising. Accordingly, we propose hypothesis 4: the positive correlation between health self-efficacy and self-rated health in Chinese college teachers is mediated by physical exercise. And hypothesis 5: the positive correlation between growth mindset of health and self-rated health in Chinese college teachers is sequential mediated by health self-efficacy and physical exercise.

## 2 Methods

### 2.1 Participants and procedures

In this study, we focused on college teachers from China, a demographic that faces unique health challenges due to the pressures of academic work and the high-stress environment associated with education (1). This choice was motivated by the observation that health outcomes among Chinese college teachers have been reported to be poorer than those of other professionals or countries, necessitating targeted research in this area (2). To ensure timely data collection, enhance response efficiency, and clarify questionnaire items, we administered face-to-face paper questionnaires. Questionnaires were distributed on-site, emphasizing their exclusive use for scientific research, confidentiality, and non-disclosure. Participants were instructed to answer honestly, with no right or wrong answers. Of the 500 questionnaires distributed, all were returned, with 456 deemed valid after excluding those with apparently consistent response or attention questions that failed the test.

Of the 456 valid questionnaires, 213 (46.71%) were from males and 243 (53.29%) from females. Majority had doctor degrees (389, 85.31%) while 67 (14.69%) had master degrees. Age distribution showed 86 (18.86%) were under 30, 219 (48.03%) were 31–40, 106 (23.25%) were 41–50, and 45 (9.87%) were over 51 years old.

### 2.2 Measures

This study employed demographic and scale measures. Gender, age, and education, potentially linked to college teachers’ health, were controlled variables. Demographic measures encompassed gender (male/female), self-reported age (integer), and education (bachelor, master, doctor). Four scales were utilized to measure key variables: growth mindset of health, health self-efficacy, physical exercise, and health.

#### 2.2.1 Growth mindset of health

Drawing inspiration from Dweck and Yeager (6), this study adapted the method of Bunda and Busseri (25) by substituting “health” for “intelligence” in the growth mindset scale to measure growth mindset of health. we adapted a first-person perspective in all items, as Zhang et al. found that first-person perspective descriptions are more effective for Chinese respondents (26).

Our scale comprised 6 items, assessing both growth mindset (e.g., “No matter what my current health is, I can change it a lot”) and fixed mindset (e.g., “My health is something about me that I cannot change very much”). Responses were scored on a 6-point Likert scale, ranging from 1 (strongly disagree) to 6 (strongly agree). Fixed mindset items were reverse-scored, then combined with growth mindset scores to calculate an average. A higher average indicated a stronger growth mindset of health. Research has shown that this scale possesses good reliability (11), and the cronbach’s α of the scale in this study was 0.90.

#### 2.2.2 Health self-efficacy

Drawing from Lee’s health self-efficacy scale (16), the Chinese adaptation exhibited solid reliability (27). This scale comprises five items, such as “I am confident I can positively influence my health.” Responses range from 1 (strongly disagree) to 5 (strongly agree) on a Likert 5-point scale. A higher average score reflected a stronger health self-efficacy. Research has shown that this scale possesses good reliability (27), and the cronbach’s α of the scale in this study was 0.76.

#### 2.2.3 Physical exercise

Drawing from the method of previous studies, physical exercise was measured through self-reported frequency. This method has proven to be an effective measure of physical exercise (13, 14). Given its long-term impact on health, participants reported their exercise frequency over the past year. They were prompted to answer, “Over the past year, how often did you exercise on average.” Responses were scored on a 7-point Likert scale, ranging from 1 (not at all) to 7 (always). A higher score reflected a more frequent exercise routine.

#### 2.2.4 Self-rated health

Drawing from the method of Bunda and Busseri (25), physical health was measured through self-rated health. This method is widely used in the “China Health and Retirement Longitudinal Study” and has proven to be an effective measure of health among Chinese people. Participants were asked to rate their current health on a 7-point Likert scale, from 1 (very poor) to 7 (very good). A higher score reflected a better physical health status.

### 2.3 Statistical analysis

We employed IBM SPSS 21 for Windows to analyze our data. Correlation analysis was performed to examine the relationship among research variables. Additionally, regression analysis was conducted to examine the potential mediation effects. These were further validated using bootstrapping through the SPSS Process Macro. All statistical significance was set at a 5% level.

## 3 Results

### 3.1 Common method variance test

The Harman one-way test was used to test for common method variance. The findings revealed four factors with characteristic roots exceeding unity. Notably, the variance attributed to the primary factor amounted to 32.43%, falling below the recommended value of 40% (28). Consequently, this study did not exhibit significant issues with common method variance.

### 3.2 Descriptive statistics and correlation analysis

Descriptive statistics and correlation analysis were computed for the study’s main variables, including means, standard deviations, and correlation coefficients, as summarized in Table 1. Notably, the variables exhibited a statistically significant positive correlation (ps < 0.01) among themselves.

**Table 1:** Descriptive statistics and correlation coefficients (*N* = 456).

Variables	*M*	*SD*	1	2	3
1. Growth mindset of health	4.77	0.85			
2. Health self-efficacy	4.12	0.51	0.49^**^		
3. Physical exercise	6.04	0.82	0.28^**^	0.42^**^	
4. Self-rated health	6.13	0.86	0.30^**^	0.52^**^	0.32^**^

^**^p < 0.01.

### 3.3 Sequential mediating effects test

**Figure 1:**
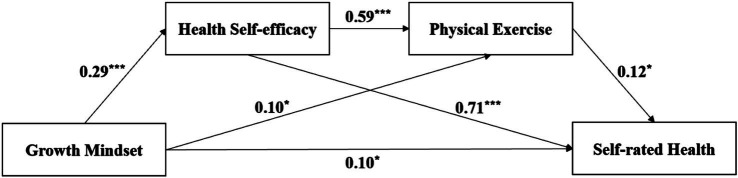
Sequential mediation model. ^*^p < 0.05, ^***^p < 0.001.

We used SPSS PROCESS Model 6 (29) to examine sequential mediating effects. Growth mindset of health served as the independent variable, while self-rated health was the dependent variable. Gender, education, and age were controlled variables. Health self-efficacy and physical exercise functioned as the first and second mediators, respectively, on the sequential mediating paths. The findings are outlined in Table 2 and Figure 1.

**Table 2:** Test of sequential mediating effects (*N* = 456).

Regression equation			Coefficient			Significance		
	Outcome variable	Predictor variable	*R*	*R* ^2^	*F*	β	*SE*	*t*
Equation 1	Self-rated health	Growth mindset of health	0.35	0.12	15.81^***^	0.34^***^	0.04	7.56
Equation 2	Health self-efficacy	Growth mindset of health	0.49	0.24	35.27^***^	0.29^***^	0.02	11.74
Equation 3	Physical exercise	Growth mindset of health	0.44	0.19	21.37^***^	0.10^*^	0.05	2.17
		Health self-efficacy				0.59^***^	0.08	7.57
Equation 4	Self-rated health	Growth mindset of health	0.54	0.29	30.82^***^	0.10^*^	0.05	2.22
		Health self-efficacy				0.71^***^	0.08	8.62
		Physical exercise				0.12^*^	0.05	2.48

^*^p < 0.05, ^***^p < 0.001.

The results showed that in the sequential mediating model, growth mindset of health positively affected health self-efficacy (β = 0.29, SE = 0.02, *p* < 0.001) and physical exercise (β = 0.10, SE = 0.05, *p* < 0.05). Furthermore, health self-efficacy positively affected physical exercise (β = 0.59, SE = 0.08, *p* < 0.001) and self-rated health (β = 0.71, SE = 0.08, *p* < 0.001). Additionally, physical exercise positively affected self-rated health (β = 0.12, SE = 0.05, *p* < 0.05).

We used the range of confidence interval of the Bootstrap test (30) to test whether the mediation effect was significant. The 95% confidence interval range for the sequential mediating effects in this study was [0.01, 0.05], indicating that this sequential mediating effect was significant. The path coefficient of growth mindset of health directly affecting self-rated health was significant, so the sequential mediating effect of growth mindset of health affecting self-rated health through health self-efficacy and physical exercise was partially mediated.

The effect sizes of the mediating pathways were calculated, revealing a 59.59% effect through health self-efficacy, a 3.42% effect through physical exercise, a 5.78% sequential effect through both, and a total mediating effect of 68.79%. These findings are summarized in Table 3.

**Table 3:** Effect sizes of mediated path (*N* = 456).

	Pathway	β	LLCI	ULCI	ES
Direct effect		0.10	0.01	0.19	
Indirect effect	Health self-efficacy	0.20	0.15	0.27	59.59%
	Physical exercise	0.01	0.01	0.04	3.42%
	Health self-efficacy and physical exercise	0.02	0.01	0.05	5.78%
	Total indirect effect	0.23	0.17	0.31	68.79%
Total effect		0.34	0.25	0.43	

## 4 Discussion

### 4.1 Theoretical significance

First of all, this study explored the relationship between growth mindset of health and self-rated health of Chinese college teachers, which enriched the knowledge of protective factors of physical health of college teachers. Prior research indicated that stress mindset among them buffered the negative impact of work stress on emotions and burnout (1). Building on this, our investigation revealed similar findings among college teachers prone to health issues. Notably, growth mindset of health emerged as a protective factor for college teachers’ physical health, echoing previous research on college students (4, 25). Our results suggest that growth mindset of health is universally beneficial, indicating its potential as a protective factor for both students and teachers.

Secondly, this study expands the mindset theory literature. Previous works of growth mindset primarily focused on educational, social, and clinical psychology. While recent literature had explored growth mindset in health psychology (10), they mainly centered on behaviors like smoking, exercise, and diet (4, 31). Little attention has been paid to the impact of growth mindset on physical health-related outcomes. However, in other domains, growth mindset has been linked to outcomes like academic performance, mental health, and happiness (32–34). By focusing on health outcomes, our study fills a crucial gap in mindset theory, particularly in the health field. We found a positive link between growth mindset of health and physical health, indicating that individual growth beliefs about health positively predict related outcomes, similar to how growth mindset predicts other areas of success (32–34).

Thirdly, this study deepens our understanding of self-regulation theory. Utilizing this framework, we examined the sequential mediating roles of health self-efficacy and physical exercise in explaining the positive relationship between growth mindset and health among college teachers. This exploration fills a theoretical gap by clarifying how growth mindset facilitates individuals’ self-regulation of health. Specifically, college teachers with stronger growth mindset exhibited higher health self-efficacy, enhancing their self-regulation abilities. These teachers also engaged more in physical exercise, actively participating in the self-regulation process. Furthermore, a higher sense of health self-efficacy prompted increased physical exercise, suggesting that self-regulation perception precedes the self-regulation process. This finding not only offers a fresh theoretical perspective for research on college teachers’ physical health but also interprets the impact of growth mindset on health outcomes through the lens of self-regulation theory (7), elucidating its mediating effects.

### 4.2 Practical significance

College teachers with stronger growth mindset of health are more confident in managing their own health and actively engage in physical exercise, resulting in better health outcomes. Therefore, human resource departments and labor unions in colleges and universities should incorporate the development of teachers’ growth mindset of health and self-efficacy into their health-promoting plans, alongside regular physical check-ups and health education. Conversely, teachers with weaker mindset are less active and unhealthier. College leaders should identify these teachers and implement targeted interventions to enhance their health self-control, encouraging physical activity and improved health. Lastly, as exercise is integral to health, universities should organize sports events and competitions to foster teachers’ enthusiasm for physical activity and thereby promote their overall health.

### 4.3 Limitations and future prospects

Firstly, we used a cross-sectional survey, showing positive correlations but not causation. Future researches should consider longitudinal studies or experimental designs to establish causal relationships between growth mindset of health and physical health outcomes.

Secondly, the sample was limited to Chinese college teachers, which may restrict the generalizability of findings. Future studies should include diverse populations to assess the influence of demographical factors on growth mindset of health. Additionally, while the measures used showed adequate reliability, further validation in various cultural contexts is necessary to ensure their robustness.

Thirdly, this study’s data solely relied on college teachers’ self-reports, introducing methodological limitations. To enhance accuracy, future studies should incorporate reports from family or colleagues and consider objective measures, such as physiological data from wearable devices or regular health assessments.

Lastly, while this study explored the mediating roles of health self-efficacy and physical exercise, it focused specifically on these dimensions of self-regulation. Future research could investigate additional self-regulation mechanisms (e.g., self-control, goal-setting) and consider alternative theoretical frameworks, such as conservation of resource theory (35), to broaden understanding of growth mindset.

## 5 Conclusion

Using self-regulation theory, this study initially examined how college teachers’ growth mindset of health related to their self-rated health, focusing on the mediating roles of health self-efficacy and physical exercise. Results indicated a positive correlation between growth mindset and health, with health self-efficacy and physical exercise serving as sequential mediators. Specifically, college teachers with stronger growth mindset reported higher health self-efficacy, leading to increased physical exercise and better health. Future researches could employ experimental and longitudinal methods, include diverse populationsm, and use objective measures to further validate these findings. Additionally, exploring mechanisms from alternate perspectives would be beneficial.

## Data Availability

The raw data supporting the conclusions of this article will be made available by the authors, without undue reservation.
